# Risk factors for the severity of Guillain-Barré syndrome and predictors of short-term prognosis of severe Guillain-Barré syndrome

**DOI:** 10.1038/s41598-021-91132-3

**Published:** 2021-06-02

**Authors:** Puyuan Wen, Lisha Wang, Hong Liu, Li Gong, Han Ji, Hongliang Wu, Wenzheng Chu

**Affiliations:** Department of Neurology, The affiliated Yantai Yuhuangding Hospital of Qingdao University, Yantai, 264000 Shandong People’s Republic of China

**Keywords:** Neurology, Risk factors

## Abstract

Guillain-Barré syndrome (GBS) is a neurological disorder characterized by paralysis. Identifying the severity, appropriate therapeutic method, and prognosis of GBS at an early stage is highly important. This study aimed to investigate the modifiable risk factors for the severity of GBS and consequent need for mechanical ventilation (MV) and to identify clinical predictive factors for poor short-term outcomes of severe GBS. 155 GBS patients who were admitted to the Affiliated Yantai Yuhuangding Hospital of Qingdao University during 2014–2020 were enrolled. Demographic, clinical, therapeutic and evolutionary data were collected and were then analyzed using univariate and multivariate regression analyses. Our analytic data demonstrated that the significant clinical predictors of severe GBS were recent history of surgery, older age, cranial nerve impairment, and elevated levels of liver enzymes (*p* < 0.05). Furthermore, autonomic dysfunction, lower Medical Research Council (MRC) score at nadir, and elevated levels of liver enzymes were significantly associated with MV for severe GBS (*p* < 0.05), and lower MRC score at nadir and autonomic dysfunction remained significant predictors of MV in severe GBS (*p* < 0.05). Lastly, recent history of surgery, lower MRC score at admission and at nadir, requirement for MV, and pneumonia during hospitalization were significantly associated with the short-term outcome of severe GBS and that lower MRC score at admission and need for MV were confirmed to be predictors of poor short-term prognosis (*p* < 0.05). Of note, this study suggested that recent history of surgery is a predictor of severity in GBS patients and is associated with the poor short-term prognosis of severe GBS.

## Introduction

Guillain-Barré syndrome (GBS) is an inflammatory demyelinating polyradiculoneuropathic condition^1^. In the acute phase, this condition is characterized by generalized paralysis, bulbar muscle weakness, autonomic dysfunction, respiratory failure, presence or absence of sensory association with hyporeflexia or areflexia, and absence of cerebrospinal fluid pleocytosis.

Approximately 30% of GBS patients have respiratory failure; therefore, they require endotracheal intubation and mechanical ventilation (MV) support^1,2^. Thus, respiratory failure is a life-threatening manifestation, which is the leading cause of death among GBS patients^3^. Furthermore, severe GBS patients require close monitoring in intensive care unit (ICU), as well as the need for artificial ventilation to save life. Therefore, there is an urgent need for identifying severity at an early stage and formulating proper guidelines for allocating GBS patients to a suitable department (common ward or the ICU) to decrease the incidence of respiratory distress and mortality.

A remarkable prognostic factor of severe GBS is the need of MV^4^. Therefore, it is highly important to identify patients who may require intubation and MV upon hospital admissions. The clinical features at the onset of GBS are diverse. Patients with severe GBS are likely to develop serious complications including pneumonia and sepsis which could lead to a poor prognosis^5^. In order to ameliorate the prognosis of GBS patients who also experienced respiratory failure, it is important to identify symptoms using predictors and promptly apply appropriate medical interventions to GBS patients with the risk of developing into a severe disease stage.

In previous studies, the number of patients diagnosed with severe GBS, especially those undergo respiratory failure, is generally low. To overcome this problem, we conducted a retrospective study on patients with severe GBS. We identified multiple clinical risk factors that contribute to the disease progression of GBS, the possibility of developing respiratory failure and the potential of poor prognosis. Our study provides valuable information that may help reduce the poor prognosis of patients with severe GBS and insights for broader applications both clinically and financially.

## Results

### Demographic features of GBS patients

**Figure 1:**
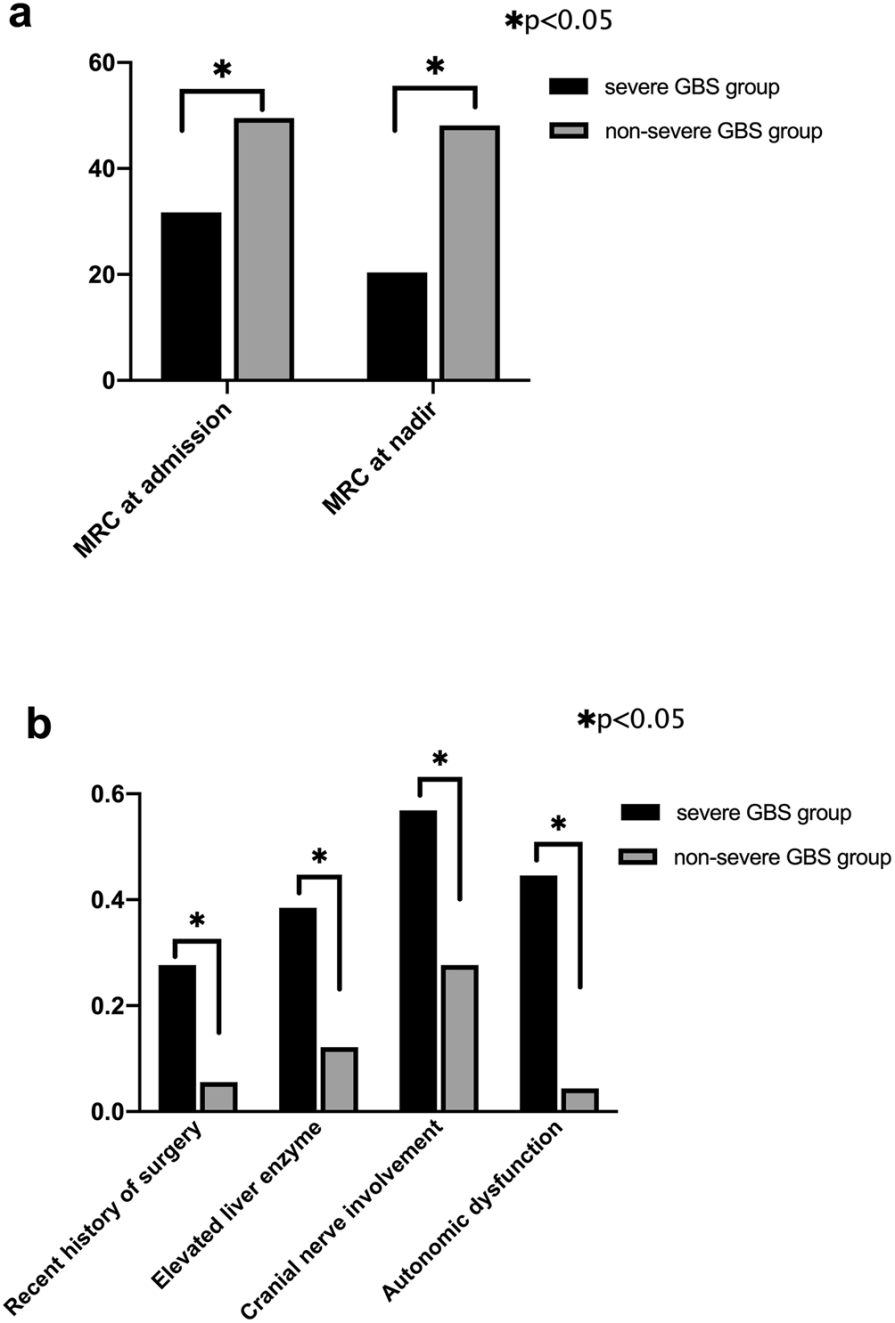
Comparisons between severe GBS group and non-severe GBS group. (**a**) MRC at admission and MRC at nadir in severe GBS group were both lower than non-severe GBS group (both *p* < 0.05). (**b**) In severe GBS group, recent history of surgery, elevated liver enzyme, cranial nerve involvement and autonomic dysfunction were totally higher than non-severe GBS group(*p* < 0.05).

A total of 155 patients with GBS were enrolled in present study. The average age of onset was 56.15 ± 15.81 years, and the majority were men (57.4%). Twenty-one patients had post-surgical GBS, and the surgeries that tended to complicate GBS were, in descending order, neurosurgery, orthopedic surgery, gastrointestinal surgery, vascular, and cardiovascular surgery (Table 1). Sixty-five (41.9%) patients with HFGS score ≥ 4 points at nadir were classified into the severe GBS group, and the remaining 90 patients were classified into the non-severe GBS group. Differences between the severe GBS and non-severe GBS groups are shown in Table 2. We demonstrated that the severe GBS group had older patients than the non-severe GBS group (60.6 years versus 52.9 years, *p* < 0.05). As shown in Fig. 1a, the MRC scores at admission and at nadir were both lower in the severe GBS group (31.7 versus 49.5 and 20.4 versus 48.1, both *p* < 0.05). As indicated in Fig. 1b, cranial nerve impairment and autonomic dysfunction were both more common in the severe GBS group than in the non-severe GBS group (56.9% versus 27.7% and 44.6% versus 4.4%, both *p* < 0.05). In the severe GBS group, a recent history of surgery and elevated levels of liver enzymes were both higher than those in the non-severe GBS group (24.6% versus 5.6% and 38.5% versus 12.2%, *p* < 0.05). In addition, pneumonia occurred more frequently after admission in GBS patients (49.2% vs. 2.2%, *p* < 0.05), and these patients had a shorter time from symptom onset to hospital admission (5.4 days versus 9.6 days, *p* < 0.05). Sex, season of morbidity, and place of residence were not significantly different between the groups (*p* > 0.05), and the same as tendon reflex, sensory dysfunction, and pain.

**Table 1:** Details of surgeries.

Surgery	N (Total of n = 21)
Neurosurgery	13 (61.9%)
Brain glioma resection	2
Intracranial hematoma removal	4
Intracranial aneurysm surgery	2
Brain trauma surgery	5
Gastrointestinal surgery	2 (9.5%)
Appendectomy	2
Orthopedic surgery	3 (14.3%)
Discectomy	2
Ankle arthroplasty	1
Vascular surgery	2 (9.5%)
Lower extremity varicose vein surgery	1
Inferior vena cava filter placement	1
Cardiovascular surgery	1 (4.8%)
Percutaneous coronary intervention (PCI)	1

**Table 2:** Comparison of clinical characteristics and presentation of GBS between severe GBS and non-severe GBS groups.

Variable	Severe GBS group (n = 65)	Non-severe GBS group (n = 90)	*p*
Age (years)	60.6 ± 14.9	52.9 ± 15.7	< 0.0001
Male sex	33 (50.8)	56 (62.9)	0.155
Place of residence			0.107
Urban community	26 (40.0)	49 (54.4)	
Countryside	39 (60)	41 (45.6)	
Incidence of GBS in different seasons			0.348
Spring	16 (24.6)	15 (16.7)	
Summer	21 (31.8)	28 (31.1)	
Autumn	12 (18.5)	27 (30.0)	
Winter	16 (24.6)	20 (22.2)	
Antecedent infection	19 (39.2)	31 (34.4)	0.278
Sensory disturbance	16 (24.6)	35(38.9)	0.09
Pain	7 (10.8)	12 (13.3)	0.631
Hyporeflexia or areflexia	62 (95.4)	83 (92.2)	0.85
Time from onset to hospital admission	5.4 ± 8.2	9.6 ± 7.8	0.002
Treatment modality			0.109
IVIg	53(81.6)	73(81.1)	
IVIg + intravenous Corticosteroids	8(12.3)	5(5.6)	
Intravenous corticosteroids	1(1.5)	7(7.7)	
Supportive treatment	3(4.6)	5(5.6)	
Complicated by pneumonia	32 (49.2)	29 (2.2)	0.0001

GBS: Guillain-Barré syndrome; IVIg: Intravenous immunoglobulin.

### Clinical risk factors for severity

Univariate logistic regression analysis revealed that age, recent history of surgery, autonomic dysfunction, cranial nerve impairment, elevated levels of liver enzymes, and MRC sum score on admission and at nadir were significantly different between the severe GBS and non-severe GBS groups (*p* < 0.05). Furthermore, older age, cranial nerve impairment, elevated levels of liver enzymes, and recent history of surgery were the significant clinical risk factors of severe GBS in multivariate analysis (*p* < 0.05) (Table 3).

**Table 3:** Possible independent risk factors for the severity of GBS in multivariate logistic regression.

Variable	Regression coefficient (95%) CI	*p*	Exp (B)
Recent history of surgery	1.758 (1.857–18.131)	0.012	5.803
Cranial nerve involvement	0.228 (1.031–1.529)	0.023	1.256
Elevated liver enzymes	1.419 (1.733–9.849)	0.001	4.132
Age	0.033 (1.007–1.061)	0.012	1.034

GBS: Guillain-Barré syndrome; CI: Confidence interval.

### Clinical prognostic factors for MV in the group with severe GBS

**Figure 2:**
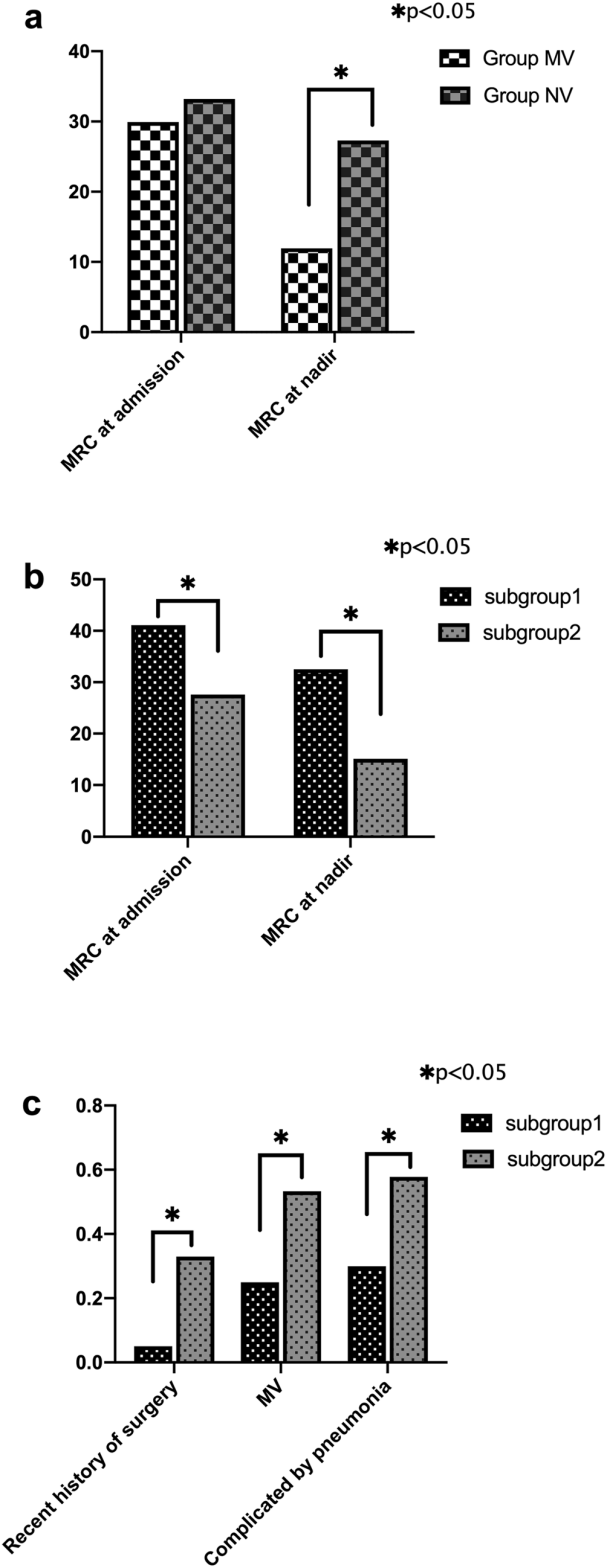
Comparisons between Group MV and Group NV, Subgroup 1 and Subgroup 2. (**a**) MRC at nadir in Group MV were lower than Group NV (*p* < 0.05), but MRC at admission had no difference between Group MV and Group NV in statistic. (**b**) In Subgroup 1, MRC at admission and at nadir were both higher than Subgroup 2(both *p* < 0.05). (**c**) Recent history of surgery, requiring MV and complicated by pneumonia were totally lower in Subgroup 1 than Subgroup 2(*p* < 0.05).

For 65 patients with severe GBS, the average disease onset age was 60.5 ± 15.4 years old and the majority patients were males (65.5% vs. 38.5%, *p* < 0.05). The MV group has 29 GBS patients that administrated with MV. The NV group contained 36 severe GBS patients without administration of MV. These two groups were compared and summarized in Table 4. Based on this information, we found that there was no significant difference (*p* > 0.05) in the season of morbidity, residence location, patient surgical history, MRC score on admission between MV and NV groups. Patients in these two groups also did not significantly differ in tendon reflex, scales of pain and sensory dysfunction. Notably, the MRC score at nadir of patients in MV group was significantly lower than that in NV group (11.9 vs. 27.1, *p* < 0.05, as shown in Fig. 2a). In addition, patients in MV group had increased level of liver enzymes compared with patients in NV group (65.5% vs. 16.7%, *p* < 0.05). The frequency of the involvement of cranial nerve (82.8% vs. 36.1%, *p* < 0.05) and dysautonomia (93.1% vs. 5.6%, *p* < 0.05) were both higher in patients from MV Group compared with NV group. Moreover, patients from MV group experienced significantly longer duration of hospitalization compared with those in NV group (30.1 days vs. 13.1 days, *p* < 0.05).

**Table 4:** Comparison of clinical characteristics and presentation of GBS between Groups MV and NV and between Subgroups 1 and 2.

Variable	Severe GBS patients (N = 65)					
	Group MV (n = 29)	Group NV (n = 36)	*p*	Subgroup 1 (n = 20)	Subgroup 2 (n = 45)	*p*
Age (years)	61.55 ± 15.2	59.56 ± 15.7	0.607	59.95 ± 15.8	60.7 ± 15.4	0.864
Male sex	19 (65.5)	14 (38.9)	0.059	10 (50)	23 (51.1)	0.934
Place of residence			0.839			0.583
Urban community	12 (41.4)	14 (38.9)		11 (55)	28 (62.2)	
Countryside	17 (58.6)	22 (61.1)		9 (45)	22 (37.8)	
Incidence of GBS in different seasons			0.967			0.077
Spring	8 (27.6)	8 (22.2)		5 (25)	11 (24.4)	
Summer	9 (31)	12 (33.3)		6 (30)	15 (33.3)	
Autumn	5 (17.2)	7 (19.4)		7 (35)	5 (11.1)	
Winter	7 (24.1)	9 (25.0)		2 (10)	14 (31.1)	
Antecedent infections	10 (34.5)	9 (25)	0.384	9 (45)	12 (26.7)	0.927
Recent history of surgery	7 (24.1)	9 (25)	0.936	1 (5)	15 (33.3)	0.014
Cranial nerve involvement			0.002			0.697
Facial nerve	10 (34.5)	6 (16.7)	0.097	5 (25)	11 (24.4)	0.962
Glossopharyngeal and vagus nerves	12 (41.4)	7 (19.4)	0.053	5 (25)	14 (31.1)	0.617
Oculomotor and/or abducent nerve	7 (24.1)	4 (11.1)	0.164	3 (15)	8 (17.8)	0.783
Sensory disturbance	6 (20.7)	10 (27.8)	0.51	5 (25)	11 (24.4)	0.962
Autonomic dysfunction	27 (93.1)	2 (5.6)	< 0.001	7 (35)	22 (48.9)	0.298
Elevated liver enzymes	19 (65.5)	6 (16.7)	< 0.001	7 (35)	18 (40)	0.702
Pain	1 (3.4)	6 (16.7)	0.087	3 (15)	4 (8.9)	0.463
Hyporeflexia or areflexia	27 (93.1)	35 (97.2)	0.694	17 (85)	39 (86.7)	0.304
Time from onset to hospital admission	4.1 ± 4.5	6.4 ± 10.26	0.27	6.5 ± 6.4	4.9 ± 8.9	0.497
Hospital stay	30.1 ± 21.5	13.1 ± 6.3	0.001	17.15 ± 8.9	22.2 ± 19.7	0.157
Treatment modality						0.362
IVIg				16 (80)	38 (84.5)	
IVIg + intravenous Corticosteroids				3 (12.5)	4 (8.9)	
Intravenous corticosteroids				1 (5)	0	
Supportive treatment				0	3 (6.7)	

GBS: Guillain-Barré syndrome; MV: Mechanical ventilation; IVIg: Intravenous immunoglobulin.

Analysis of univariate logistic regression showed that MV was positively correlated with dysautonomia, lower MRC score at nadir, and increased level of liver enzymes (*p* < 0.05). With analysis of multivariate logistic regression, we showed that lower MRC score at nadir and dysautonomia were predisposing factors for GBS patients that require MV (*p* < 0.05), as shown in Table 5.

**Table 5:** Possible independent predictors for MV and poor short-term outcome in severe GBS by multivariate logistic regression.

Variable	Regression coefficient (95%) CI	*p*	Exp (B)
**Predictors for MV**			
MRC at nadir	− 0.123 (0.795–0.984)	< 0.05	0.885
Autonomic dysfunction	6.429 (619.568–17,111.894)	< 0.05	619.568
**Predictors for poor short-term outcome**			
MRC at admission	− 0.054 (0.909–0.988)	< 0.05	0.948
Requiring MV	1.461 (1.146–16.224)	< 0.05	4.312

GBS: Guillain-Barré syndrome; MV: mechanical ventilation; MRC: Medical Research Council; CI: Confidence interval.

### Clinical predictors for poor short-term outcome prognosis in the group with severe GBS

A total of 65 severe GBS patients were divided into two groups. In Subgroup 1 there were 20 patients with good prognosis, and Subgroup 2 included 45 patients with poor outcomes. The comparison between these two groups was summarized in Table 4. In particular, Subgroup 1 has significantly higher scores of MRC at nadir and at admission compared with that in Subgroup 2 (32.5 versus 15.1, and 41.1 versus 27.6, both *p* < 0.05, Fig. 2b). Patients in these two groups had no significant difference in the timeframe from symptom onset to hospital admission and seasonal pattern of morbidity (*p* > 0.05). However, patients in Subgroup 2 had a higher frequency of recent surgical history than Subgroup 1 patients (33.3% vs. 5%, *p* < 0.05, as shown in Fig. 2c). In addition, patients in Subgroup 1 had significantly lower requirement for MV compared with Subgroup 2 patients (25% vs. 53.3%, *p* < 0.05, Fig. 2c). Given that hospital-acquired pneumonia could impact on the prognosis of patients with severe GBS, we compared the incidence rate of pneumonia between these two groups. We found that the incidence of pneumonia was higher in Subgroup 2 than in Subgroup 1 (57.8% vs. 30%, *p* < 0.05, Fig. 2c). In addition, using univariate logistic analysis, we showed that a recent surgical history, lower MRC scores on admission and at nadir, treatment with MV, and complications such as pneumonia were significantly correlated with poor prognosis of GBS patients (*p* < 0.05). Moreover, analysis of multivariate logistic model showed that lower score of MRC at nadir and the requirement with MV were two individual prognostic factors for poor disease outcomes (*p* < 0.05, as shown in Table 5).

## Discussion

In our study, we investigated predictors of severity in GBS patients at early stages, requirement of MV, and short-term outcomes in severe GBS. The predictive factors for severe GBS included older age, recent history of surgery, cranial nerve impairment, and elevated levels of liver enzymes. The predictors for the requirement of MV in severe GBS patients included lower MRC at nadir and autonomic dysfunction. For prognostic factors in severe GBS patients, we confirmed that lower MRC scores at admission and at nadir, requiring MV, and complications with pneumonia were associated with poor short-term prognosis, and lower MRC score at admission and requiring MV were found to be predictors of poor short-term prognosis. We believe that these results may help clinicians to assign GBS patients to the appropriate unit, to decide whether or not tracheotomy and ventilator-assisted ventilation should be performed, to assess the prognosis, and to develop a clinical prediction model.

Even though GBS is an acute, self-limiting autoinflammatory disease, the majority of GBS patients recover completely or with minor sequelae^6^. However, patients with severe GBS are frequently suffered from unfavorable residual sequelae or even mortality^7^; Meanwhile, many hospitals have insufficient medical resources and limited number of intensive care units. Therefore, it is important to identify patients with severe GBS at the early stage of disease and assign them with intensive care units to reduce the occurrence of residual sequelae and mortality. However, few studies have assessed the severity of GBS. In our study, the interval time from symptom onset to hospital admission and MRC scores at admission and at nadir were associated with severe GBS in univariate logistic regression; they were not continuously associated with severe GBS in multivariate logistic regression. In addition, we found that older age, cranial nerve impairment, and elevated levels of liver enzymes were correlated with the severity of GBS in multivariate logistic regression. It was reported that intravenous immunoglobulin (IVIg) therapy could elevate liver enzymes transitorily through an unknown mechanism^8^, and patients with GBS have transient mild liver dysfunction with undetermined etiology^8,9^; Furthermore, elevated liver enzymes could be caused by intraoperative use of anesthetics and drugs^10,11^; In our study, the significant differences in elevated levels of liver enzymes between severe group and non-severe group may be due to severe GBS itself and surgery, the specific reasons and mechanisms need further study. Of note, a recent history of surgery was the most significant predictor of severe GBS in the present study. Patients with a recent history of surgery were 5.8 times more likely to develop severe GBS than those without a history of surgery. The main types of surgeries included neurosurgery, orthopedic surgery, gastrointestinal surgery, vascular surgery, and cardiovascular surgery. Some studies^12,13^ demonstrated that patients with GBS induced by surgery presented with severe movement disorder and respiratory failure, which was in accordance with the results of our study. The potential pathophysiological mechanisms of post-surgical GBS are not yet clearly understood. Clinical and/or subclinical infections secondary to post-surgical short-term immunosuppressive conditions has been reported to induce GBS^14^. Additionally, the breakdown of the innate protective barrier causes antigens present in the blood to enter the nervous system during surgery, thereby allowing antigens to initiate the subsequent autoimmune responses^15^. Nonetheless, further research is needed to clearly clarify the pathogenesis.

It is of prime importance to predict the need for MV at the early stage because 60% of GBS patients with MV experienced many complications that increase the risk of mortality; thus, early recognition and intervention of GBS may decrease the occurrence of complications and ameliorate its prognosis^16–18^. Heterogeneous studies have been conducted to investigate the predictive factors of the need for MV in GBS patients. The present study found two predictors for the requirement of MV in severe GBS patients. One was autonomic dysfunction; we found that severe GBS patients with dysautonomia were more likely to need MV than those without autonomic dysfunction. This was in accordance with the results of a cohort study that reported dysautonomia as an independent predictor of respiratory insufficiency^19,20^ and the results of Bangladesh et al. that identified bulbar involvement, autonomic dysfunction, and severe muscle weakness as important risk factors for MV among Bangladeshi GBS patients^21^. Therefore, strengthening airway management is very important for GBS patients with autonomic dysfunction. The other predictor for the requirement of MV was lower MRC score at nadir, which was verified in multivariate analysis to be the predictor for requiring MV. Islam et al. found that severe muscle weakness (MRC sum scores ranging from 0 to 20) at study entry was more likely to progress to MV^21^. The NSB score model developed by Kanikannan and colleagues could accurately predict the requirement of MV by single breath count, neck weakness, and bulbar palsy^22^.

In our study, only 30.8% of severe GBS patients had good short-term prognosis. Because of its negligible neurological sequelae, early identification of predictors for prognosis in severe GBS patients may ameliorate their outcome and improve their quality of life. Walgaard et al.^23^ established a modified Erasmus GBS Outcome Score to predict the outcome of GBS at 6 months, which includes MRC sum score at admission and on the seventh day, age, and history of diarrhea. Gonzalez-Suarez et al.^24^ demonstrated older age, severe deficits at onset, cranial nerve involvement, requiring MV, and axonal lesion patterns in the NCS as poor prognostic factors for GBS. Further, Netto et al.^25^ showed that older age, dysautonomia, and pulmonary complications served as predictors of mortality in MV patients with GBS. The absence of antecedent infections and lower MRC sum score at nadir have been reported to be predictors of poor short-term prognosis in mechanically ventilated GBS patients^7^. However, few studies have investigated the predictors of prognosis for severe GBS. In our study, requiring MV and MRC at admission were found to be predictors of prognosis for severe GBS. Additionally, we found that a recent history of surgery, lower MRC at nadir, and complications with pneumonia were associated with poor short-term outcomes of severe GBS by univariate logical analysis. However, the results were not observed in the multivariate analysis, which may be due to the small sample size. Of note, a recent history of surgery was not a predictor for the severity of GBS alone; it may also be associated with the poor prognosis of severe GBS, although we found that recent surgery was irrelevant to the requirement of MV. From the above, it is reasonable to speculate that surgery preceding GBS may affect the limb muscles more than the respiratory muscles, and further research is needed to confirm this wherein the topic will be in depth in the following days. When patients develop progressive muscle weakness rapidly after surgery, GBS should be considered.

There are also limitations in our study. First, our study used retrospective analysis which was designed in a monocentric manner: the prognosis was performed predominantly on the hospitalized patients and lacked follow-up observations, making it impossible to analysis the long-term prognosis of GBS. Second, due to the nature of retrospective research, clinical indexes, including various species of IgG antiganglioside antibodies, vital capacity, electrophysiological recordings, which were proposed to be important risk factors leading to MV treatment could not be collected. Third, this study failed to collect detailed information on the involvement of autonomic nervous system and complications in patients treated with MV. Lastly, the sample size in this study is unsatisfactory for analysis in a stratified manner and requires further prospective studies to confirm our findings.

### Conclusions

In our study, clinical risk factors of severity in GBS, the requirement of MV, and unfavorable short-term prognosis in severe GBS have been clearly expressed in our study. A recent history of surgery is a predictor of severity in GBS patients and is associated with the poor short-term prognosis of severe GBS, but further research is needed to confirm this and be in depth.

## Methods

### Ethical approval

This retrospective study complied with the Declaration of Helsinki and its amendments and was approved by the Ethics Committee of the Affiliated Yantai Yuhuangding Hospital of Qingdao University (Yantai, China). Because of the retrospective nature of our study, the need for informed consent was waived.

### Study design and setting

All methods were performed in accordance with the relevant guidelines and regulations by including a statement in the methods section to this effect. Subjects were selected among patients who met the diagnostic criteria of GBS^26^ and received sequential therapy during hospitalization in the Department of Neurology of Yuhuangding Hospital Affiliated to Qingdao University between January 2014 and July 2020. Subjects were excluded from the study if they were aged < 18 years, refused treatment, or were diagnosed with either bickerstaff encephalitis, critical illness polyneuropathy/myopathy, chronic inflammatory demyelinating polyradiculoneuropathy^27^, or Miller Fisher syndrome. We also excluded patients who were hospitalized for ≤ 3 days because their disease severity might not have reached the worst condition when discharged and they might have lost the data. Clinical data from all subjects were analyzed retrospectively, including information on age, sex, place of residence (urban community or countryside), season of disease occurrence, history of antecedent infections (mainly diarrhea and upper respiratory tract infection), recent history of surgery (GBS symptom onset within 6 weeks), time from onset to hospital admission, hospital length of stay, clinical severity evaluated by the Hughes Functional Grading Scale (HFGS) score at nadir/admission, muscle strength assessed based on the Medical Research Council (MRC) sum score at nadir/admission, tendon reflex, cranial nerve damage (including glossopharyngeal and vagus nerves, facial nerve, oculomotor and/or abducent nerve), sensory disturbance, whether or not MV is needed, autonomic nerve dysfunction, abnormal hepatic enzyme, therapeutic method, and complications during hospitalization (mainly pneumonia). Autonomic nerve dysfunction included cardiovascular autonomic nervous dysfunction (systolic blood pressure change over 40 mmHg), spontaneous severe bradycardia (heart rate decreased > 20 times/min) or spontaneous tachycardia (heart rate > 120 times/min without fever), abnormal sweating, abnormal pupil, and urinary and stool dysfunction. Sensory disturbance included subjective numbness or pain in the limbs or lower back, objective hypoesthesia or hypersensitivity, and deep sensory disturbance. Abnormal hepatic enzyme levels were defined as abnormal levels of aspartate aminotransferase and/or alanine aminotransferase, which were 1.5 times higher than the normal value on the second day after admission. Moreover, an HFGS score ≥ 4 points at the nadir was regarded as severe GBS^28^.

### Assessment of neurological functional deficit and clinical severity

All 155 patients were assessed for neurological functional impairment and clinical severity. The HFGS was applied to evaluate functional impairment, which has 6 degrees^29^: 0, normal; 1, mild symptoms and able to run; 2, capable of walking > 5 m without assistance from others but cannot run; 3, capable of walking > 5 m with assistance; 4, chairbound or bedridden; 5, requiring MV for breathing; and 6, dead. Additionally, the MRC score, for which the total score ranges from 0 to 60, was used to evaluate muscle strength. The score was calculated according to the strength in six bilateral muscles in the four limbs^30^. The lowest MRC score or the highest HFGS score was defined as the nadir of GBS.

### Assessment of short-run outcome and grouping

All 155 patients were divided into groups of two on the basis of HFGS: severe GBS group (HFGS ≥ 4)^28^ and non-severe GBS group (HFGS < 4). In the severe GBS group, 66 patients were further divided into two subgroups depending on the requirement for MV or not: Group MV included GBS patients requiring MV, and Group NV included GBS patients not requiring MV at the nadir of illness. Generally, the patient was discharged from the hospital when the condition improved or was stable in our department. Additionally, in this study, patients who could walk with assistance when discharged (HFGS ≥ 3) were judged to have a favorable short-term outcome. In contrast, patients who could not walk even with assistance (HFGS < 3) were considered to have a poor short-term outcome. In view of the above, severe GBS patients were further divided into two subgroups: Subgroup 1, patients with good short-term outcomes, and Subgroup 2, patients with poor short-term outcomes.

### Statistical analyses

In the present study, SPSS, version 17.0, software and GraphPad Prism 8 were used for all statistical analyses. Categorical data were expressed as proportions and tested using the *Chi-s*quare test. All continuous data were accorded with normal distribution and were expressed as means ± standard deviations and tested using independent *t*-tests. Univariate logistic regression analysis was used to evaluate the independent predictors of severity of GBS, the requirement of MV and unfavorable short-term outcomes in severe GBS. Variables that were significant in univariate analysis were further analyzed in multivariate regression analysis. For all statistical tests, *p* < 0.05 was deemed to indicate statistically significant difference.

### Ethics approval and consent to participate

This study was approved by the ethics committee of Yuhuangding Hospital Affiliated to Qing University, because of its retrospective nature, all participants did not provide written informed consent.

## Data Availability

The datasets analyzed during the current study are available from the corresponding author on reasonable request.
